# Feasibility study of transfer function model on electrocardiogram change caused by acupuncture

**DOI:** 10.1186/s12906-017-1615-5

**Published:** 2017-02-08

**Authors:** Haebeom Lee, Hyunho Kim, Jungkuk Kim, Hwan-Sup Oh, Young-Jae Park, Young-Bae Park

**Affiliations:** 1 Department of Human Informatics of Korean Medicine, Interdisciplinary Programs, Kyung Hee University, Seoul, South Korea; 2 Department of Biofunctional Medicine & Diagnostics, College of Korean Medicine, Kyung Hee University, Seoul, South Korea; 3 Department of Electronics Engineering, Myongji University, Gyeonggi-do, South Korea; 4 Department of Mechanical Engineering, Kyung Hee University, Gyeonggi-do, South Korea

**Keywords:** Acupuncture, Transfer function, ECG, TCM, Korean medicine

## Abstract

**Background:**

Acupuncture treatments that regulate the heart are used to treat various clinical disorders and conditions. Although many studies have been conducted to measure quantitatively the effects of acupuncture, thus far, models that describe these effects have not been established. The purpose of this study was to derive a transfer function model of acupuncture stimulation within the electrocardiograms based on the periods before, during, and after acupuncture.

**Methods:**

Fourteen healthy subjects were included in this clinical trial. Five-minute electrocardiograms were captured before, during, and after acupuncture at HT7. For each period, signal-averaged electrocardiograms were created from all of the subjects’ 5-min electrocardiograms for that period. Individual transfer functions, which has the highest average goodness of fit, were derived for each period pair. By averaging individual transfer functions, generalized transfer functions were derived.

**Results:**

The transfer function with the highest average goodness of fit was a fraction with 4th order numerator and 5th order denominator. Fourteen individual transfer functions were derived separately for each pair of periods: before and during acupuncture, during and after acupuncture, and before and after acupuncture. Three generalized transfer functions were derived by averaging individual transfer functions for each period pair.

**Conclusion:**

The three generalized transfer functions that were derived may reflect the electrocardiogram changes caused by acupuncture. However, this clinical trial included only 14 subjects. Further studies with control groups and more subjects are needed.

This clinical trial has been registered on the Clinical Research Information Service, Republic of Korea (No. KCT0001944). The first enrolment of subject started at 2 June 2015 and this trial was retrospectively registered at 14 June 2016

## Background

Acupuncture can have local, segmental, extrasegmental, and central regulatory effects [1]. Thus, acupuncture is known to affect not only the somatic nervous system but also the autonomic nervous system (ANS). Acupuncture can be used to treat various clinical disorders or conditions related to the ANS, such as insomnia [2] and hypertension [3]. HT7 (*Shenmen*) is a commonly used acupuncture point used to treat these symptoms [4–6].

To measure effects of acupuncture quantitatively, many studies were conducted by using blood tests, positron emission tomography (PET) [7, 8], functional MRI (fMRI) [9–12], or heart rate variability (HRV) [13, 14]. Acupuncture can reduce heart rate and change HRV by activating the parasympathetic nervous system in the ANS [15, 16]. However, these previous studies lacked quantitative assessments and specific models to describe changes.

In mechanical engineering and electrical engineering, when a known system model is not available, a system identification method is often used. Since transfer function (TF) is a well-known system identification technique to derive a model from an input and output signal, it is widely used in automobile engineering [17], acoustics engineering [18], and electronic circuit engineering [19]. In the biosignal field, TF models are used to analyze EEGs and vibration systems in vehicles [20], human acoustic systems [21], and vascular systems [22–24]. However, much is still unknown about the physiological mechanism or model of electrocardiogram (ECG) changes caused by acupuncture. Therefore, a mathematical model cannot be derived from the previous studies, and simulation of ECG after acupuncture stimulation is not possible.

In this study, to derive a mathematical model, we applied an acupuncture treatment at HT7 (*Shenmen*) on the dominant hand and conducted 5-min ECGs before, during, and after acupuncture to the healthy subjects. After individual TF (ITF) models were derived from each pair of periods, generalized TF (GTF) models were derived by averaging all subjects’ ITFs for each pair of periods. By this method, ECG changes caused by acupuncture could be expressed as a GTF. If the error of the GTF and the ITF are similar, the acupuncture stimulus, as well as the personal characteristics, may have a specific effect on the ECG. Therefore, GTF is a mathematical model that can reflect the characteristics of acupuncture stimulation. We try to develop a mathematical model that reflects the general characteristics of acupuncture stimulus by the clinical trial.

## Methods

### Subjects

All subjects participated voluntarily. The inclusion criteria were as follows: (1) age between 20 and 49 years; (2) ability to communicate adequately about his or her physical condition with a clinical researcher and fill out the questionnaires; and (3) willingness to participate in this clinical trial and sign a written informed consent form. The following subjects were excluded from the study: (1) subjects having an abnormal heart beat period; (2) subjects practicing Qigong or who were athletes; (3) subjects with a cardiovascular disease history including hypertension, arrhythmia, or ischemic heart disease; (4) subjects who had taken cardiovascular-related medicines in the past month; and (5) subjects judged inappropriate by the researcher.

**Fig. 1:**
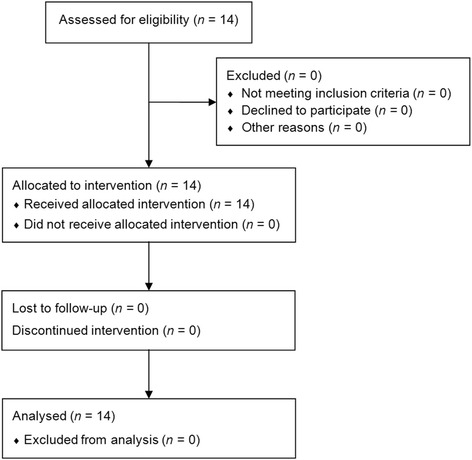
CONSORT flowchart of the clinical trial

From June 2 to 25 in 2015, 14 subjects were recruited; none was excluded. Sample size 14 was decided by 12 participants from “rule of 12” with 20% marginal window that considering possible drop out [25]. Ultimately, 14 healthy subjects were included in this clinical trial (4 males, 10 females; age, 24–32 years; mean age, 27.07 ± 2.67 years). All subjects were allocated to single intervention group and there is no drop-out subject. Data from all subject were used for this study. The CONSORT flowchart of this trial is shown in Fig. 1.

### ECG instrument and questionnaire

We used an LXC3203 (Laxtha, INC., Daejon, South Korea) and Telescan (Laxtha Inc., Daejon, Republic of Korea) to measure a 4-lead ECG. The sampling frequency was 1024 Hz. Only the lead II signal from the ECG was analyzed in this study.

Subjects were asked to fill out the Korean language version of the Massachusetts General Hospital Acupuncture Sensation Scale (MASS) questionnaire [26] and assess their sensations with the intensity of *de qi* scale [27]. *De qi* is the sensation caused by acupuncture and is an important aspect of acupuncture treatment [26]. To confirm that *de qi* occurred during the acupuncture treatment, we used the MASS index from MASS questionnaire and the scores from the intensity of *de qi* scale. The MASS questionnaire has 13 questions about sensations during the needle-manipulating phase and stationary phase. The intensity of *de qi* scale is a visual analogue scale for *de qi* intensity. One value was missing for one subject; we substituted it with the mean of the other subjects’ responses.

### Procedure

**Fig. 2:**
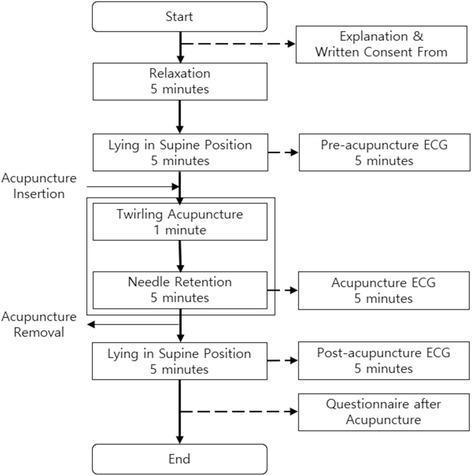
Procedure of the clinical trial

The procedure of this trial is shown in Fig. 2. Before each subject was included in this clinical trial, we received written consent from him or her after he or she had received a written description of this trial. The clinical trial was conducted in a quiet, designated room at Kyung Hee University Korean Medicine Hospital. ECGs were obtained from the standard 4 leads on the extremities.

After 5 min of relaxation in a comfortable supine position, the pre-acupuncture ECG was recorded for another 5 min. The acupuncture ECG was recorded for 5 min after inserting and twirling an acupuncture needle for 1 min at HT7 of subject’s dominant hand. After withdrawing the acupuncture needle, post-acupuncture ECG was recorded for 5 min. After the withdrawal of acupuncture needle, subjects were asked to fill out the MASS questionnaires and *de qi* intensity questionnaire. After a doctor of Korean medicine confirmed that the subject did not have bleeding or other side effects, that subject was considered to have completed the trial. Incentives was given after the completion of the trial.

This clinical trial was approved and supervised by Kyung Hee University Korean Medicine Hospital’s Institutional Review Board (No. KOMCIRB-150420-HR-015). The authors confirm that all ongoing and related trials for this intervention are registered.

### Acupuncture intervention

All of the acupuncture treatments were performed by the same doctor of Korean medicine, who had 2 years’ clinical experience. Subjects received treatment at HT7 (*Shenmen*) on the dominant hand. HT7 is a commonly used acupuncture point for observing HRV [28, 29]. The location of HT7 was determined by using the general guidelines in the World Health Organization Standard Acupuncture Point Locations in the Western Pacific Region [30]. We selected the dominant hand for the acupuncture treatment to be consistent with previous acupuncture studies [31, 32]. The skin near the HT7 acupuncture point was sterilized with 79% alcohol before needle insertion. A 0.30 × 40 mm acupuncture needle (DongBang Medical, Gyeonggi-do, South Korea) was inserted to a depth of 5–10 mm with a disposable guide tube (DongBang Medical, Gyeonggi-do, South Korea). After the needle was inserted, the guide tube was removed. The twirling method was used to generate a needle sensation. The needle was manipulated for 1 min with 2 Hz and then retained for 5 min without any stimulation. Immediately after 5 min of needle retention, the needle was withdrawn.

### Data analysis

Excel 2010 (Microsoft, Redmond, WA, USA) and SPSS Statistics 18 (SPSS, Inc., Chicago, IL, USA) were used for data preprocessing and statistical calculations. Matlab R2015a (Mathworks, Inc., Natick, MA, USA) was used to analyze signal processing.

The lead II signal from the standard 4-lead ECG was used. Signal was filtered with a 0.5–45 Hz band-pass filter. The 5-min ECG signal from lead II was divided into several single ECGs (SECGs) with average heart rate. After reviewing every SECG, all abnormal SECGs that have 2 times higher amplitude than the average SECG of the subject were removed for data cleansing. A single averaged ECG (SAECG) was derived by averaging all SECGs except abnormal SECGs and by aligning the R peaks of SECGs. Consequently, for each subject, an SAECG was obtained at 3 time points: before acupuncture, during acupuncture, and after acupuncture.

The TF, G(z), is a function that can show a relation between the input signal, X(z), and the output signal, Y(z) when z means discrete-time signal in a complex frequency domain: G(z) = Y(z)/X(z). Generally, the TF of a discrete signal comprises a fraction including the n-th order polynomial numerator and m-th order polynomial denominator when *b* is the coefficient of numerator and *a* is the coefficient of denominator (Eq. 1).

1 $$ G z=\frac{b_n{z}^n+{b}_{n-1}{z}^{n-1}+\cdots +{b}_0}{a_m{z}^m+{a}_{m-1}{z}^{m-1}+\cdots +{a}_0} $$

The TF was derived by using the tfest function in Matlab for the SAECGs of 3 pairs of periods (before and during acupuncture [BDA], during and after acupuncture [DAA], and before and after acupuncture [BAA] as the former is the input and the latter is the output) and by aligning the R peaks of the SAECGs within each pair. To use the tfest function in Matlab, m should be ≥ n [33]. As we supposed that our TF model would not have feedthrough, eventually we derived n-1th numerator order by the *tfest* function that made the leading coefficient of numerator to 0 [33].

If *y*_*m*_ is a measured signal and *y*_*s*_ is a signal simulated by TF, then the goodness of fit (GF) can be calculated by Eq. 2 using the *compare* function in Matlab [34, 35]. || indicates the 2-norm of a vector. If GF goes to 100, it means that the simulated signal has high similarity to the measured signal.

2 $$ \mathrm{Goodness}\ \mathrm{of}\ \mathrm{Fit}=100\times \left(1-\frac{\left\Vert {y}_m-{y}_s\right\Vert }{\left\Vert {y}_m-\overline{y_m}\right\Vert}\right) $$

For the best fit, we explored the GFs of ITFs with 1st–5th order polynomial denominators to find a simple model. An ITF was derived for each subject and for each BDA, DAA, and BAA; the GF for each ITF was then determined. After collecting every GF of ITFs for each period pair, the order of the numerator and the order of the denominator that have highest average GF were used to determine the optimized order. A GTF was derived by averaging each subject’s ITF on this optimized order [36, 37]. The process of averaging TF is to average every subject’s coefficient on each order and on a specific period pair.

## Results

### *De qi* intensity

The MASS index results were as follows: the average was 6.2 out of 10; the standard deviation (SD) was ±1.80; the minimum was 2.48; and the maximum was 8.49. The results for *de qi* intensity were as follows: the average was 5.6 out of 10; the SD was ±1.76; the minimum was 2.0; and the maximum was 8.0. These results indicate that sufficiently intense *de qi* sensations were provided in this trial.

### Goodness of fit from all subjects

Table 1 shows the average GFs from all subject’s GFs for 1st-5th denominator order of TF for each period pair. The TF with 4th order polynomial numerator and 5th order polynomial denominator has the highest average GF, 94.48. The average GF seems to be saturated under 95, 97, and 94, respectively.

**Table 1:** Averaged goodness of fit for each transfer function for each subject

ON/OD	GF of BDA	GF of DAA	GF of BAA	Mean GF
0/1	92.48	94.07	90.64	92.40
0/2	87.72	95.77	91.46	91.65
1/2	93.56	95.83	92.05	93.81
0/3	87.84	89.12	82.69	86.55
1/3	94.21	96.25	92.35	94.27
2/3	93.43	96.38	92.78	94.20
0/4	42.37	53.79	45.00	47.05
1/4	84.56	86.03	77.63	82.74
2/4	87.84	95.35	92.87	92.02
3/4	93.85	96.21	93.05	94.37
0/5	16.59	30.57	16.97	21.38
1/5	76.49	80.97	48.41	68.62
2/5	83.13	96.41	87.08	88.87
3/5	92.01	96.26	88.18	92.15
4/5	94.91	95.73	92.81	94.48

*ON* order of numerator, *OD* order of denominator, *GF* goodness of fit, *BDA* periods before and during acupuncture, *DAA* periods during and after acupuncture, *BAA* periods before and after acupuncture

### GTFs and ITFs

**Fig. 3:**
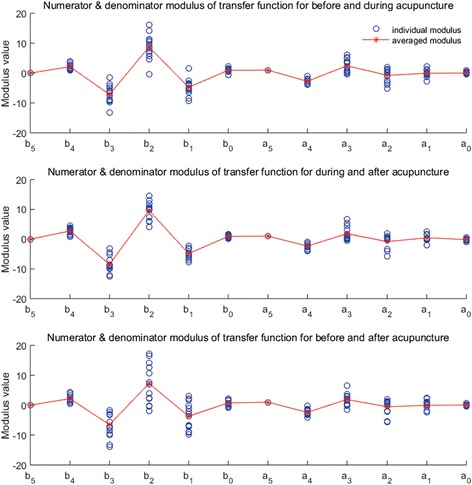
Coefficients of numerator and denominator of generalized transfer function

Each order’s coefficient of every subject’s ITFs (circle) and GTF (star and line) for BDA, DAA, and BAA pairs are shown in Fig. 3.

G_BD_(z) is the GTF for BDA, G_DA_(z) for DAA, and G_BA_(z) for BAA. The functions showed as Eqs. 3, 4, and 5.

3 $$ {G}_{BD}(z)=\frac{2.1655{z}^4-7.1340{z}^3+8.7569{z}^2-4.7618 z+0.9737}{z^5-2.7147{z}^4+2.4578{z}^3-0.7302{z}^2-0.0416 z+0.0292} $$

4 $$ {G}_{DA}(z)=\frac{2.7395{z}^4-8.4351{z}^3+96060{z}^2-4.8410 z+0.9312}{z^5-2.3208{z}^4+1.8338{z}^3-0.8285{z}^2+0.4626 z-0.1466} $$

5 $$ {G}_{BA}(z)=\frac{2.1861{z}^4-6.5264{z}^3+7.2857{z}^2-3.6988 z+0.7536}{z^5-2.3920{z}^4+1.9120{z}^2-0.5381{z}^2-0.0529 z+0.0713} $$

**Fig. 4:**
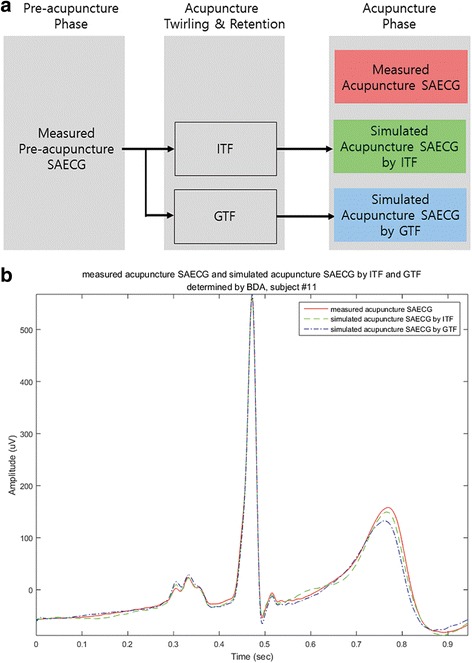
Three SAECGs and simulated results. **a** Three SAECGs; measured SAECG, simulated SAECG by individual, and generalized transfer function. **b** Example of 3 acupuncture SAECGs: a measured SAECG, a simulated SAECG by individual transfer function, and a simulated SAECG by generalized transfer function. ITF, individual transfer function; GTF, generalized transfer function; SAECG, signal-averaged electrocardiogram; BDA, before and during acupuncture

For example, Fig. 4a shows a diagram for 3 SAECGs that are used for TF of BDA in Fig. 4b. The measured pre-acupuncture ECG is used as the input signal for the ITF and GTF. The measured acupuncture SAECG is used for calculating GF. Figure 4b shows the 3 acupuncture-phase SAECGs of subject 11. The line is a measured SAECG, the dashed line is the simulated SAECG by ITF for the acupuncture phase, and the dash-dot line is the simulated SAECG by GTF for the acupuncture phase. In the ideal case, the 3 SAECGs would be identical, but differences were seen between the measured acupuncture SAECG, the acupuncture SAECG estimated by ITF, and the acupuncture SAECG estimated by GTF.

Table 2 shows GFs by ITF and GTF. The average GF differences between ITF and GTF were 0.23, 1.47, and 0.98 for before and during acupuncture, during and after acupuncture, and before and after acupuncture, respectively.

**Table 2:** Goodness of fit of individual and generalized transfer function model

	Before and during acupuncture		During and after acupuncture		Before and after acupuncture	
ID	GF of ITFM	GF of GTFM	GF of ITFM	GF of GTFM	GF of ITFM	GF of GTFM
1	94.03	94.35	97.95	95.34	95.62	94.47
2	97.53	95.90	94.71	92.98	90.41	90.27
3	98.01	97.23	94.19	93.59	93.78	92.49
4	98.44	98.03	97.96	97.33	98.15	97.85
5	96.16	94.30	97.68	96.44	94.75	91.71
6	96.06	95.71	98.31	97.79	96.03	95.62
7	90.90	88.93	90.89	88.10	82.35	76.95
8	92.65	92.94	97.16	96.57	95.66	93.88
9	93.02	91.76	96.68	96.02	91.02	88.24
10	83.03	96.42	98.48	97.72	94.01	94.43
11	92.95	87.80	97.65	96.82	91.32	84.31
12	92.42	90.16	96.93	96.60	94.03	93.50
13	94.47	93.76	97.51	97.44	92.98	92.02
14	94.03	93.19	98.91	98.60	89.08	92.88
Avg	93.84	93.61	96.79	95.81	92.80	91.33
SD	3.857	3.074	2.157	2.712	3.894	5.282

*ID* subject identification number, *GF* goodness of fit, *ITFM* individual transfer function model, *GTFM* generalized transfer function model, *Avg* average, *SD* standard deviation

## Discussion

### Transfer function analysis

To quantify the ECG changes caused by acupuncture, we considered the system identification method. In the field of biomedical engineering, not only is TF model used, but also impulse response model [38] and state space model [39] are widely used as well. The advantage of the TF model is that the system can be easily expressed. Because of this, we chose to use the TF model over other system identification methods.

The previous studies in which the TF model was used addressed the phase of TF [24, 40]. We did not derive the phase of TF, because the SAECG is not a periodically repeated signal, unlike ECGs used in the previous studies [36, 37]. What we have derived is the TF for an ECG magnitude change from SAECG for a certain period to the SAECG for another period. For example, the acupuncture SAECG can be predicted when pre-acupuncture SAECG is used as the input signal for the TF of BDA.

We derived 3 GTFs from the SAECG changes caused by acupuncture stimulation. These mathematical functions indicate that acupuncture can cause changes in the heart’s electrical system, and they explain the general ECG changes in subjects as well. These 3 GTFs demonstrate different phenomena. In this clinical trial, the TF of BDA reflects acupuncture insertion, twirling, and retention. The TF of DAA reflects acupuncture twirling, retention, and removal. The TF of BAA reflects acupuncture insertion, twirling, and retention. In future studies, 1 or 2 TFs related to a specific type of stimulation can be derived from the result of pairing 2 periods (BDA, DAA, or BAA).

Because this study was a feasibility study, we tried to find the differences and tendencies among the 3 GTFs. We tried to find the same optimized TF order that has the highest GF under the order to be ≤ 5th order denominator for the 3 GTFs. To find the optimized order, we made 15 TFs and calculated 15 GFs per subject and period pair (Table 1). For 14 subjects and 3 period pairs, 630 TFs were needed. To find the optimized TF order ≤ 10th order denominator, we need 2310 TFs. Since 2310 TFs can be too large to probe, we had limited the maximum degree of denominator to 5th order. In this study, we decided 4th order numerator and 5th order denominator as the optimized order, because that TF with optimized orders has the highest average GF under the limit we set.

If a further study were carried out only for a specific period pair, the optimized order might be different. For example, the optimized order for only a BAA is a 3th order numerator and 4th order denominator.

### Further study and limitations

From these TFs, we derived models of ECG changes caused by acupuncture stimulation. From these TFs, a SAECG during acupuncture or after acupuncture can be predicted from an arbitrary SAECG before acupuncture, and a SAECG after acupuncture can be predicted from an arbitrary SAECG during acupuncture with a high GF. In previous studies, signal changes caused by acupuncture were hard to predict. HRV was mainly analyzed statistically and it can be influenced by individual differences in the ANS and daily activity patterns [41]. Among the many studies using ECG, there have been a lot of studies that used statistical methods to test the difference of the changes, but the methods of quantitatively predicting the changes were rarely used. The method we used can be used to predict a post-stimulation signal from a specific model with a high GF. In addition 5-min SAECG is widely used because it is also robust against small errors by averaging the signals while 20-min of ECG recording recommended for HRV [42–44].

In this study, we found that the TF method can represent the difference between before, during, and after acupuncture stimulation as a mathematical function. Based on this, it is possible to identify quantitative differences between mathematical models of acupuncture points after finding the mathematical function of ECG changes by acupuncture points in acupuncture stimulation. In addition, the change of ECG according to the needle stimulation technique can be quantitatively expressed as the difference of the mathematical function, so that a quantitative comparison can be made as to which technique changes a lot of ECG. The optimal acupuncture points and techniques for ECG and autonomic control can be found and used in clinically. In addition, this method can be used to make a model that explains the signal changes between a pre-intervention and a post-intervention period. As a result, this method can be useful to evaluate and quantify acupuncture interventions, because an exclusive or singular model related for acupuncture has not established yet.

In the aspect of study design, this study does not include any control group, but only compare the ECG signals before, during, and after acupuncture. There is a disadvantage that it is not known what type of needle stimulation due to the ECG, i.e. whether it is specific to HT7 or needle twirling.

This study was a feasibility study, and the sample size was 14 subjects. Because of this small size, the GTF can be sensitive to outlier subject. To generalize our results, a larger sample size and comparative studies are needed, but we tried to find a feasibility of TF model for acupuncture in this study.

## Conclusions

There is no known system model that can describe the ECG changes caused by acupuncture stimulation. We took ECGs before, during, and after acupuncture. For each subject, we made 3 SAECGs: 1 for each period. ITFs were derived from BDA, DAA, and BAA SAECG pairs. By averaging the ITFs, GTFs were derived for each period pair. As a result of prediction of BDA, DAA and BAA with GTF, average GF was 93.6, 95.8, 91.3. The GTF was slightly lower than the ITF, but the difference was small. This means that acupuncture stimulation has a common characteristic to the ECG, and it can be represented by this mathematical model.

## Abbreviations

ANS: Autonomic nervous system
BAA: Before and after acupuncture
BDA: Before and during acupuncture
DAA: During and after acupuncture
ECG: Electrocardiogram
fMRI: Functional MRI
GF: Goodness of fit
GTF: Generalized TF
HRV: Heart rate variability
ITF: Individual TF
MASS: Massachusetts General Hospital Acupuncture Sensation Scale
PET: Positron emission tomography
SD: Standard deviation
SECG: Single ECG
TF: Transfer function

## References

[1] White A, Cummings M, Filshie J (2008). An introduction to western medical acupuncture, 1e.

[2] Shergis JL, Ni X, Jackson ML, Zhang AL, Guo X, Li Y (2016). A systematic review of acupuncture for sleep quality in people with insomnia. Complement Ther Med.

[3] Liu Y, Park J-E, Shin K-M, Lee M, Jung HJ, Kim A-R (2015). Acupuncture lowers blood pressure in mild hypertension patients: a randomized, controlled, assessor-blinded pilot trial. Complement Ther Med.

[4] Yeung W-F, Chung K-F, Poon MM-K, Ho FY-Y, Zhang S-P, Zhang Z-J (2012). Prescription of Chinese herbal medicine and selection of acupoints in pattern-based traditional Chinese medicine treatment for insomnia: a systematic review. Evid-Based Complement Altern Med Evid-Based Complement Altern Med.

[5] Cevik C, Işeri SO (2013). The effect of acupuncture on high blood pressure of patients using antihypertensive drugs. Acupunct Electrother Res.

[6] Chae Y, Park HJ, Kang OS, Lee HJ, Kim SY, Yin CS, Lee H (2011). Acupuncture attenuates autonomic responses to smoking-related visual cues. Complement Ther Med.

[7] Scheffold BE, Hsieh C-L, Litscher G (2015). Neuroimaging and neuromonitoring effects of electro and manual acupuncture on the central nervous system: a literature review and analysis. Evid Based Complement Alternat Med.

[8] Shen J (2001). Research on the neurophysiological mechanisms of acupuncture: review of selected studies and methodological issues. J Altern Complement Med N Y N.

[9] He T, Zhu W, Du S-Q, Yang J-W, Li F, Yang B-F (2015). Neural mechanisms of acupuncture as revealed by fMRI studies. Auton Neurosci.

[10] Chae Y, Chang DS, Lee SH, Jung WM, Lee IS, Jackson S, Kong J, Lee H, Park HJ, Lee H, Wallraven C (2013). Inserting needles into the body: a meta-analysis of brain activity associated with acupuncture needle stimulation. J Pain.

[11] Huang W, Pach D, Napadow V, Park K, Long X, Neumann J, Maeda Y, Nierhaus T, Liang F, Witt CM (2012). Characterizing acupuncture stimuli using brain imaging with FMRI--a systematic review and meta-analysis of the literature. PLoS One.

[12] Chae Y (2012). Acupuncture and brain imaging: what do we have to consider?. Acupunct Med.

[13] Lee S, Lee MS, Choi J-Y, Lee S-W, Jeong S-Y, Ernst E (2010). Acupuncture and heart rate variability: a systematic review. Auton Neurosci.

[14] Chung JWY, Yan VCM, Zhang H (2014). Effect of acupuncture on heart rate variability: a systematic review. Evid Based Complement Alternat Med.

[15] Wang J, Kuo TB, Yang CC (2002). An alternative method to enhance vagal activities and suppress sympathetic activities in humans. Auton Neurosci.

[16] da Silva MAH, Dorsher PT (2014). Neuroanatomic and clinical correspondences: acupuncture and vagus nerve stimulation. J Altern Complement Med.

[17] Thiene M, Ghajari M, Galvanetto U, Aliabadi MH (2014). Effects of the transfer function evaluation on the impact force reconstruction with application to composite panels. Compos Struct.

[18] Elliott S, Stothers I, Nelson P (1987). A multiple error LMS algorithm and its application to the active control of sound and vibration. IEEE Trans Acoust Speech Signal Process.

[19] Yin Y, Zane R, Glaser J, Erickson RW (2003). Small-signal analysis of frequency-controlled electronic ballasts. IEEE Trans Circuits Syst Fundam Theory Appl.

[20] Broman H, Pope M, Hansson T (1996). A mathematical model of the impact response of the seated subject. Med Eng Phys.

[21] Aibara R, Welsh JT, Puria S, Goode RL (2001). Human middle-ear sound transfer function and cochlear input impedance. Hear Res.

[22] O’Rourke MF, Avolio AP (2008). Arterial transfer functions: background, applications and reservations. J Hypertens.

[23] Meel-van den Abeelen ASS, van Beek AHEA, Slump CH, Panerai RB, Claassen JAHR (2014). Transfer function analysis for the assessment of cerebral autoregulation using spontaneous oscillations in blood pressure and cerebral blood flow. Med Eng Phys.

[24] Chen C-H, Nevo E, Fetics B, Pak PH, Yin FCP, Maughan WL (1997). Estimation of central aortic pressure waveform by mathematical transformation of radial tonometry pressure validation of generalized transfer function. Circulation.

[25] Charity M, Rickey C, Paul N, Paul S (2011). Recommendations for planning pilot studies in clinical and translational research. Clin Transl Sci.

[26] Kong J, Gollub R, Huang T, Polich G, Napadow V, Hui K (2007). Acupuncture *De Qi*, from qualitative history to quantitative measurement. J Altern Complement Med.

[27] Oh HJ, Lee ES, Lee YJ, Lee SD, Kim KS, Kim EJ (2013). The clinical study about qualitative and quantitative characteristics of acupuncture sensation according to the body parts. The Acupuncture.

[28] Huang H, Zhong Z, Chen J, Huang Y, Luo J, Wu J (2015). Effect of acupuncture at HT7 on heart rate variability: an exploratory study. Acupunct Med.

[29] Litscher G, Liu C-Z, Wang L, Wang L-P, Li Q-Q, Shi G-X (2013). Improvement of the dynamic responses of heart rate variability patterns after needle and laser acupuncture treatment in patients with burnout syndrome: a transcontinental comparative study. Evid Based Complement Alternat Med.

[30] WHO western pacific region (2008). WHO standard acupuncture point locations in the western pacific region. WHO standard acupuncture point locations in the western pacific region.

[31] Pfab F, Huss-Marp J, Gatti A, Fuqin J, Athanasiadis GI, Irnich D (2009). Influence of acupuncture on type I hypersensitivity itch and the wheal and flare response in adults with atopic eczema - a blinded, randomized, placebo-controlled, crossover trial: effect of acupuncture on allergen-induced itch in atopic eczema. Allergy.

[32] Xu M, Zhou S-J, Jiang C-C, Wu Y, Shi W-L, Gu H-H (2012). The effects of P6 electrical acustimulation on postoperative nausea and vomiting in patients after infratentorial craniotomy. J Neurosurg Anesthesiol.

[33] Mathworks, Transfer function estimation. http://www.mathworks.com/help/ident/ref/tfest.html. Accessed 4 Apr 2016.

[34] Mathworks, Compare model output and measured output. http://www.mathworks.com/help/ident/ref/compare.html. Accessed 15 Dec 2016.

[35] Mathworks, Goodness of fit between test and reference data. http://www.mathworks.com/help/ident/ref/goodnessoffit.html. Accessed 4 Apr 2016.

[36] Saul JP, Berger RD, Albrecht P, Stein SP, Chen MH, Cohen RJ (1991). Transfer function analysis of the circulation: unique insights into cardiovascular regulation. Am J Physiol.

[37] Berger RD, Saul JP, Cohen RJ (1989). Transfer function analysis of autonomic regulation. I. Canine atrial rate response. Am J Physiol.

[38] Ehlert FA, Korenstein D, Steinberg JS (1997). Evaluation of P wave signal-averaged electrocardiographic filtering and analysis methods. Am Heart J.

[39] Fojt O, Holcik J (1998). Applying nonlinear dynamics to ECG signal processing. Two approaches to describing ECG and HRV signals. IEEE Eng Med Biol Mag Q Mag Eng Med Biol Soc.

[40] Karamanoglu M, O’Rourke MF, Avolio AP, Kelly RP (1993). An analysis of the relationship between central aortic and peripheral upper limb pressure waves in man. Eur Heart J.

[41] Grossman P (2004). Respiratory sinus arrhythmia, cardiac vagal control, and daily activity. AJP Heart Circ Physiol.

[42] Schneider MAE, Plewan A, Schmitt C, Meinertz T (1996). The signal-averaged ECG obtained by a New digital holter recording system. Ann Noninvasive Electrocardiol.

[43] Goldberger JJ, Challapalli S, Waligora M, Kadish AH, Johnson DA, Ahmed MW (2000). Uncertainty principle of signal-averaged electrocardiography. Circulation.

[44] Electrophysiology TF o. t. ES o. C t. NAS (1996). Heart rate variability : standards of measurement, physiological interpretation, and clinical use. Circulation.

